# Progress toward poliomyelitis eradication in Kano State, Nigeria, 2010 - 2017

**DOI:** 10.11604/pamj.supp.2021.40.1.19318

**Published:** 2021-11-12

**Authors:** Suleiman Haladu Ahmed, Patrick Nguku, Saheed Oluwatoyin Gidado, Musa Kalamullah Bawa, Usman Lawan Shehu, Amina Abdullahi, Ramatu Usman Obansa, Kabir Ibrahim Getso, Mohammd Nasir Mahmoud, Imam Wada Bello, Yahaya Musa Sharif, Bashir Abba, Sani Umar, Ndadilnasiyya Endie Waziri, Chima Ohunabunwo

**Affiliations:** 1 Field Epidemiology Network, Abuja, Nigeria,; 2 Ministry of Health, Kano, Nigeria,; 3 World Health Organization, Kano Office, Nigeria,; 4 Morehouse School of Medicine, Atlanta GA, USA

**Keywords:** Kano State, wild polio virus, polio eradication

## Abstract

**Introduction:**

Kano State in Northern Nigeria was a major source of Wild Polio Virus (WPV) cases in Nigeria up until 2015. In 2009, the State reported 168 WPV cases out of the 388 reported nationally. This paper characterizes the progress made by Kano State in polio eradication.

**Methods:**

In December 2017, we conducted a descriptive review of Routine Immunization (RI) from both the District Vaccine Data Management Tool (DVD-MT) and District Health Information System (DHIS2) from 2010 to 2017. Also, we reviewed the Acute Flaccid Paralysis (AFP) and Supplementary Immunization Activities (SIAs) data reported for Kano State from 2010 to 2017. Also, we obtained the number of reported WPV cases by serotypes.

**Results:**

From 2010 to 2017, a total of 65 confirmed WPV cases were reported in Kano State. Of these, 58 (89%) were WPV1 and 7 (11%) WPV3. Almost half of these cases were reported in 2012 from 14 LGAs. The number of reported cases fell to 15 (23%) in 10 LGAs in 2013, and further decreased to 5 (8%) in four LGAs in 2014. No new WPV cases have been detected in Kano since 2015. During the same period, 23 circulating Vaccine Derived Polio Viruses (cVDPV2) cases were reported in Kano. Specifically, 10 LGAs reported 10 cases in 2011. Three LGAs reported three cases in 2012, while eight LGAs reported 10 total cases in 2014. During the 2010 to 2017 period 61 SIAs were conducted.

**Conclusion:**

Kano State made progress toward polio eradication. Sustained eradication efforts, in form of high quality RI, SIAs and AFP surveillance are necessary to avert possible importation from 2016 polio resurgence in nearby Borno State, Nigeria.

## Introduction

In 1988, the World Health Assembly resolved to eradicate poliomyelitis [1]. In 2012, the Nigerian government activated an emergency operations center and implemented a national emergency action plan to eradicate polio [2]. From 2012 to 2015, Nigeria witnessed drastic reduction in the number of WPVs, from 122 cases reported in 2012 to six in 2014, [3]. The six wild poliovirus type 1 (WPV1) cases in 2014 were geographically limited to five in Kano and one in Yobe state [3]. After twenty-three months without detection of WPV1, four cases were detected in Borno state [4-6]. In 2013, Boko Haram insurgency resulted in the decline in SIA performance in Kano State resulting in suspension of the SIA in February 2013 after murders of health care workers and cancellations of March 2013 SIAs. Later, Polio elimination efforts resumed in April 2013 health workers were apprehensive and services were provided with minimal visibility. This report summarizes the poliomyelitis eradication program (PEI) progress in Kano State from 2010-2017 and reports the epidemiology of WPV cases in the state. It also describes the Global Polio Eradication Initiative (GPEI) activities and provides recommendations to sustain the interruption of WPV transmission.

## Methods

**Study population and setting:** Kano State is one of the 36 states in Nigeria, located in the North West geopolitical zone. The state has 44 Local Government Areas (LGAs), 484 wards and 27,233 settlements. The majorities of the population are Hausa/Fulani and are predominantly Muslims. Kano is the hub for access to other cities and states in the North. The state had an estimated population of 12.1 million as per the 2016 projection of the 2006 Population Census, of which 536,459 are younger than 12 months. Administrative RI coverage rates for 2017 were 82% for BCG, 93% for Penta 3, 94% for OPV3, 93% for IPV, and 80% measles antigens.

**Study design:** a descriptive epidemiological review was conducted in December 2017. Data describing WPV and cVDPV cases reported in Kano State for the period between 2010 and 2017 were reviewed and analyzed.

**Data sources:** we extracted data from the Acute Flaccid paralysis (AFP) surveillance database housed in the epidemiology unit of the Kano State Ministry of Health, as well as records of RI from District Vaccine Data Management Tool (DVD-MT) and District Health Information System, version 2 (DHIS2) data base domiciled at World Health Organization and Monitoring and Evaluation (M&E) unit of Kano State Primary Health Care Management Board (SPHCDA). SIA data archived at the Kano EOC was abstracted. We reviewed the DVDMT data from 2010 to 2013 and abstracted annualized OPV3 and Diphtheria, Pertussis, Tetanus 3 (DPT3)/ Pentavalent 3 (Penta3) coverage rates for Kano State. Due to introduction of in DHIS2 RI module in 2014, annualized OPV 3 and Penta3 coverage rates from 2014 to 2016 were abstracted from DHIS2 platform. Cleaned AFP datasets from 2010 to 2017 were merged in Microsoft Excel Office 2010 and exported to Epi-Info statistical software, version 3.3, for analysis. Univariate analysis was performed to generate yearly distribution of WPV and cVDPV. We generated frequencies for age, gender, number of OPV doses administered and geographic distribution of WPV and cVDPV cases. Yearly trend of AFP cases reported, polio compatible cases reported and other key surveillance performance indicators. We summarized the environmental surveillance data to generate yearly trend of Polio virus isolates by serotypes from 2010 to 2017. Using the SIA data, we summarized annual number of SIAs, number of children immunized and number of OPV used from 2010 to 2017.

**Funding:** this work was funded by African Field Epidemiology Network (AFENET) through National Stop Transmission of Polio (NSTOP).

## Results

Routine immunization activities: administrative routine immunization coverage of infants less than 1 year with three doses of OPV in Kano State was 47% in 2010. This value decreased to 39% in 2011 and 2012. Afterwards the coverage increased 62% in 2013. From 2014 to 2015 there were increase to 97% and 94% respectively. Afterwards the values peaked to 112% and 101% in 2016 and 2017 (Table 1).

**Table 1:** performance of routine immunization services, Kano State, 2010 to 2017

Year	Target population of children 0-11 Month	Immunization coverage (OPV3) (administrative)	Immunization (coverage survey) DPT3/penta 3
2010	395,345	47	NA
2011	408,391	39	NA
2012	421,868	39	NA
2013	435,790	62	21.1*
2014	450,027	97	22.2
2015	465,373	94	19.0
2016	480,373	112	NA
2017	536,459	94%	16**

* Smart survey ** NICs/MICs

SIA activities: from 2010 to 2017, house to house SIAs in Kano State targeted children 0-59 months. After the global switch in April 2016, the state used bivalent (type 1 and 3) OPV exclusively. During this period approximately 310 million doses of OPV were administered during 68 SIAs and seven mop-ups as part of the outbreak response activities. In addition, Injectable Inactivated Polio Vaccine (IPV) was first introduced in December 2014 in 13 very high risk LGAs of Kano state. For this IPV SIAs, only children 3-59 months were considered, and 753,152 were immunized. In March 2015, another IPV SIA was conducted in the eight densely populated metropolitan LGAs of Kano, and 861,174 children were immunized (Table 2).

**Table 2:** number of SIAs conducted and number of OPV doses administered per round, Kano State, 2010 to 2017

Year	Number of SIAs	Number of Mop-ups	No of <5yrs immunized (M)	Number of vaccine doses used in million	Proportion of LGAs that reported >80% coverage by LQAS
2010	7	0	43. 52	49.98	NA
2011	9	0	52.07	60.45	NA
2012	7	0	36.96	40.14	12.8
2013	8	1	43.88	56.76	6.8
2014	9*	5	31.18	44.68	1.7
2015	8*	0	26.17	29.78	3.7
2016	7	1	23.29	28.61	1.2
2017	6	0	19.15	20.35	0
Total	61	7	257.07 **	310.40 **	

*IPV was introduced in the campaign for the first time in November 2014 across 13 very high risk LGAs and in March 2015 in the 8 densely populated metropolitan LGAs **Some children would have been vaccinated in multiple rounds

AFP surveillance: during 2010 to 2017, a total of 7,314 AFP cases were reported in Kano State. The AFP detection rate increased from 483 cases in 2010 to 1,409 cases in 2017. The stool adequacy rate target of 80% was achieved in all the years. In 2010, the stool adequacy rate was 94%, and increased to 97% in 2017. The non-Polio AFP rate (NP AFP) increased from nine in 2010 to 26 in 2017 among children younger than 15 years. The prevalence of non-polio enteroviruses (NPENT) isolation also increased from 166 in 2010 to 398 in 2017. Also, the number of polio compatible cases increased from 3 in 2010 to 7 in 2016 (Table 3).

**Table 3:** performance of key Acute Flaccid Paralysis (AFP) surveillance system standard indicators, Kano State, northern Nigeria, 2010 to 2017

Year	Total AFP reported	Total Polio compatible	No. of NPENT	Stool adequacy (%)	Non Polio AFP rate	NPENT rate
2010	483 (7%)	3 (9%)	166	94	9.5	*
2011	472 (7%)	7 (20%)	143	83	8.4	15
2012	473 (7%)	1 (3%)	194	88	8.2	21
2013	523 (7)	5 (14%)	170	88	9.0	16
2014	725 (10%)	5 (14%)	*	94	12.4	24
2015	1,400 (19%)	5 (14%)	419	94	23.3	15
2016	1,829 (25%)	7 (20%)	486	97	29.5	15
2017	1,409 (19%)	2 (6%)	398	97	26.4	14
Total	7,314	35	1,976	92	15.8	15

* missing data Expected rate of nonpolio AFP is > 3 case/100,000 populations < 15 years old

Environmental surveillance: the sewage sites from 2011 to 2017 indicated that 268 isolates were detected by the laboratory. In 2011, there were 25 viruses isolated, of which 13 (52%) were Sabin while 12 (48%) were VDPV2. In 2012, thirty-five virus types were detected of which 26(74%) were Sabin viruses, 1(3%) was cVDPV2 another 1 (3%) had wild poliovirus type 3, while 2 (6%) samples had wild poliovirus type 1 and 5 (14%) samples had Non polio enterovirus. In 2013, 28(70%) samples had Sabin virus, 2 (5%) samples had cVDPV2 and 10 (25%) were NPENT. In 2014, fifty-four viruses were isolated, 36 (67%) samples had Sabin, 15 (28%) cVDPV2 and 3 (6%) were NPENT. In 2015 the laboratory detected 62 isolates, 47 (76%) were Sabin and 15 (24%) NPENT. In 2016 twenty-three isolates were detected, 17(74%) were Sabin while 6 (26%) were NPENT. In 2017, there were twenty-nine isolates, 25(86%) were Sabin, while 4(14 %) were NPENT (Table 4).

**Table 4:** annual trend of polio virus isolates by serotypes from environmental surveillance sites, Kano State, Northwestern Nigeria, 2011 to 2017*

Year	WPV1	WPV3	CVDPV2	Sabin	NPENT	Total
2011	0	0	12	13	0	25
2012	2	1	1	26	5	35
2013	0	0	2	28	10	40
2014	0	0	15	36	3	54
2015	0	0	0	47	15	62
2016	0	0	0	17	6	23
2017	0	0	0	25	4	29
Total	2	1	30	192	43	268

* Environmental surveillance commenced in 2011

Epidemiology of WPV and Vaccine-Derived Poliovirus (cVDPV2): during 2010 to 2017, 65 confirmed WPV cases were reported in Kano State. Of these 58 (89%) were WPV1, while seven (11%) were WPV3. Distribution of cases by age group was as follows: children younger than 12 months constituted four (6%), children 13 to 24 months were 32 cases (49%), children 25-36 months were 16 cases (25%), children 37-48 months were five cases (8%) and children 48 months and older were eight cases (12%). Vaccination reports indicates that 19 WPV cases (29%) received zero doses OPV, 16 cases (25%) received 1-2 doses, 11 cases (17%) received 3-4 doses, 6 cases (9%) received 5-6 doses, 8 cases (12%) received more than 9 doses. The geographical distribution of WPV cases in Kano State showed that 51(78%) of all cases were found rural areas (Table 5).

**Table 5:** demographic characteristics of reported WPV and cVDPV2 cases, Kano State, 2010 to 2017

Variable	WPV1 n=58	WPV3 n=7	cVDPV2 n=23
**No of cases by year of onset**			
2010	1(2%)	0(0%)	0(0%)
2011	14(24%)	3(43%)	10(44%)
2012	23(40%)	4(57%)	3(13%)
2013	15(26)	0(0%)	0(0%)
2014	5(9%)	0(0%)	10(44%)
2015	0(0%)	0(0%)	0(0%)
2016	0(0%)	0(0%)	0(0%)
2017	0(0%)	0(0%)	0(0%)
**Demographic characteristic**			
**Age (months)**			
0 - 12	5(9%)	3(43%)	2(9%)
13 - 24	25(43%)	3(43%)	12(52%)
25 - 36	15(26%)	1(14%)	5(22%)
37 - 48	5(5%)	0(0%)	1(4%)
49 - 60	3(5%)	0(0%)	2(9%)
>60	4(7%)	0(0%)	1(4%)
**Gender**			
Male	25(43%)	3(43%)	10(43%)
Female	33(57%)	4(57%)	13(57%)
OPV coverage* in doses			
0	16(27%)	3(43%)	6(26%)
1-2	14(24%)	2(29%)	4(17%)
3-4	10(17%)	1(14%)	4(17%)
5-6	6(10%)	0(0%)	0(0%)
7-8	7(12%)	1(14%)	3(13%)
> 9	5(6%)	0(0%)	2(9%)

Annual trend of WPV and cVDPV2 cases: in 2011, the number of cases of WPV in Kano state increased sharply to 17 from one in 2010. The number of reported cases peaked in 2012 with 27 cases and started to decline to 15 in 2013 and five in 2014. This accounted for 67% decrease in number of cases. From 2015 to 2017 there was no new WPV1 case reported in Kano State. During 2010 to 2017, Kano state recorded 23 cases of cVDPV2 10 cases were reported in 2010, but the number dropped to three in 2012, the cases peaked again to 10 in 2014. However, since November 2014 no cVDPV2 were detected in any LGA of Kano State. Distribution of WPV cases by months of onset, from 2010 to 2017, shows that 40 (62%) were reported between May to August. The same trend was seen for cVDPV cases, with 16(70%) reported between May to August. The last case of WPV3, WPV1 and cVDPV2 were in September 2012, July 2014, and November 2014 respectively (Table 6).

**Table 6:** annual trend of WPV and cVDPV2 cases, Kano State, 2010 to 2017

Year	Number of WPV cases	Number of cVDPV2 cases	Number of LGAs reporting cases (WPV&cVDPV2)
2010	1	0	1
2011	17	10	14
2012	27	3	13
2013	15	0	10
2014	5	10	12
2015	0	0	0
2016	0	0	0
2017	0	0	0

## Discussion

Evidence of progress towards poliomyelitis eradication in Kano State from 2010 to 2017 include a reduction of overall WPV and cVDPV2 incidence, and decline in number of WPV isolates from environmental samples and narrowing of geographic distribution of cases. However, persistence of polio compatible cases throughout the years indicates surveillance gap and call for urgent action by the GPEI stakeholders in the State. The occurrence of polio-compatible cases indicates surveillance failure and therefore the system may not be fully relied upon to exclude with certainty the existence of areas of poliovirus transmission [7,8]. The last case of WPV 3 in Kano State was in 2012. WPV3 has not been detected in circulation since November 11, 2012 worldwide, the latest WPV3 in Africa was isolated from an infant aged 11 months in Yobe, Nigeria, who had onset of paralysis on November 10, 2012, and the latest environmental WPV3 isolate in Africa was from a sample collected in Lagos, Nigeria, on November 11, 2012 [9]. Last case of WPV1 was in 2014. The last case of cVDPV2 from an AFP case was in 2014 and last case of cVDPV2 from environmental sample was also in the same year. Although Kano State recorded all Five out of six WPV1 cases in Nigeria in 2014 [10] but, there has been a remarkable progress in reducing polio incidence by more 80% from 2012. One of the contributing factors to the progress is the improvement in the quality of routine immunization services. The highest numbers of polio cases were reported in 2011 and 2012 when the RI performance was 39% coverage by OPV3. The number of cases reduced when the RI performance increased to 97% OPV3 in 2014.

However, the quality of the administrative RI coverage is debatable due to RI 16% coverage reported by the National Indicator Survey (NICs for 2016). The introduction of IPV in the SIA in December 2014 and into the RI in March 2015 [11] might have contributed in increasing the population immunity and decline in WPV and cVDPV2. Several studies had demonstrated the use of IPV aids in preventing paralytic polio from wild or vaccine-derived type 2 polioviruses [12,13]. The quality of the SIAs improved from 2011 to 2017, this is evident by reduction in the number of LGA lots rejected to less than 80% by LQAs from 13% in 2011 to 1.2% in 2017. Periodic high-quality supplemental immunization campaigns to reach children who lack access to the routine immunization system, is one of the strategies to eradicate polio and similar infectious diseases such as measles [14].

Improvement of surveillance over the years has contributed in the progress made in polio eradication in Kano State. From the year 2010 to 2017 there was increase in AFP case detection and the two key AFP surveillance indicators have increased over the years, these key surveillance indicators are the stool adequacy and non-polio AFP rates. The NP AFP rate of ≥2/100,000 in children < 15 years is considered sensitive to detect WPV or cVDPV case if poliovirus is circulating. The second indicator is the collection of adequate stool specimen from ≥80% of patients with AFP. Adequacy refers to collection of two stool specimen ≥24 hours apart, within 14 days of paralysis onset, and arrival at a WHO-accredited laboratory in good condition [15]. Kano State had achieved both indicators over the years. However, the persistent reporting of polio compatible cases indicates surveillance gap, this underscores the need to be vigilant at enhancing the AFP surveillance in order to maintain the progress made in Polio eradication in Kano State. Environmental surveillance in Nigeria was piloted in 2011 in Kano State [16]. No WPV or cVDPV2 has been isolated since 2014. The last WPV3 and WPV1 were isolated in 2012 while the last cVDPV2 were isolated in 2014 in which 15 cVDPV 2 were isolated. These might indicate a surveillance gaps and calls for more vigilance. The finding of high number of cVDPV2 in 2014 is an indicator of low routine immunization coverage. Regular micro plan updates, and use of innovations such as the tracking of vaccination teams has help in addressing the problem of target population of children younger than 5 years.

Thanks to the efforts, the estimated target population was readjusted to three million children (from the six million projected from 2016 Census results) this correction brought about a better planned SIAs and reaching a realistic target. The quality of SIAs in Kano has greatly improved because of multiple simultaneous interventions in the form of timely release of funds, quality vaccination team selection, use of data for action and involvement of traditional and religious leaders in creating awareness about immunization and resolving noncompliance. Importantly, the introduction and scale up of Directly Observed Oral Polio Vaccination (DOPV) a vaccination strategy outside the households where male supervisors observe the administration of drops of OPV to each child, as precaution against teams who only mark the child´s finger. Other innovations health camps, (i.e. where people receive free health services for minor ailment as well as immunization for children younger than five). The systematic engagement of Quranic teachers and Nomadic population leaders popularly called Ardos, data review and harmonization at the LGA level on the 6th day of the campaign, use of polio survivor groups for immunization and communication, engagement with religious leaders, use of community clowns and local theaters for communication, and distribution of “pluses” (e.g., whistles, balloons) to attract children, tracking of Internally Displaced Persons( IDPs) all contributed to improving the quality of campaigns in the state. The massive recruitment of health care workers capacity which include WHO surge staff, cluster consultants, LGA facilitators, Field volunteers, NSTOP Local Government Officers (NSLOs), Field coordinators, deployment of Management Support Teams by AFENET, The UNICEF´s Social mobilization consultants and the Voluntary Community Volunteers (VCM) network have enforced and improve supervision and community mobilization.

Our study has two major limitations. First, the Routine Immunization performance was based on administrative coverage, due to inaccurate estimate of the target population the coverage might be overestimated. Secondly, the results we have presented in this study are based on secondary data analysis which could entail missing data and only data captured by the system, there might be other unreported cases in the community.

Although progress was made in polio eradication in Kano State, however, the risk of importation or re-introduction of poliovirus from endemic States such as Borno remains a major threat for Kano since it is a major commercial hub in Northern Nigeria with daily influx of people across the country. Moreover, insecurity and cattle rustling in some parts of Nigeria has resulted in the influx of nomadic Fulanis population to Kano. Therefore sustained AFP surveillance in children younger than 15 years is critical.

## Conclusion

Kano State has not reported any WPV or cVDPV2 cases in three years. The success recorded could be attributable to SIA, and surveillance quality occasioned by high level political and traditional leadership commitment. The establishment of EOC has provided a better coordination between Government and partners oversight to polio eradication activities at all levels.

### What is known about this topic

- Kano State is one of the high-risk states for polio transmission in Nigeria. The State was the only State that had reported all the five cases of wild polioviruses (WPVs) in Nigeria in 2014;
- The suspension of polio immunization in 2003 to 2004 due to misinformation about polio vaccine safety and of health workers deaths in 2013 has negatively impacted the performance of the SIAs and the overall polio eradication goal;
- The last WPV case was reported from Sumaila LGA of Kano in July, 2014.

### What this study adds

- Kano State has achieved progress in the polio eradication. The State has not reported any WPV or cVDPV since November, 2014;
- The establishment of EOC in 2012 resulted in the better coordination and accountability which translated in improvement in quality of immunization activities;
- Political and traditional leadership commitment and partners oversight has driven the polio program in ensuring strong coordination of government and partner at all levels.
